# Better Understanding Adult COVID-19 Vaccination Hesitancy and Refusal: The Influence of Broader Beliefs about Vaccines

**DOI:** 10.3390/ijerph19116838

**Published:** 2022-06-02

**Authors:** John Boyle, Glen Nowak, Rachel Kinder, Ronaldo Iachan, James Dayton

**Affiliations:** 1 ICF International, Rockville, MD 20850, USA; john.boyle@icf.com (J.B.); ronaldo.iachan@icf.com (R.I.); james.dayton@icf.com (J.D.); 2 Center for Health & Risk Communication, College of Journalism and Mass Communication, University of Georgia, Athens, GA 30602, USA; gnowak@uga.edu

**Keywords:** survey research, vaccine hesitancy, attitudes and beliefs, COVID-19, nonprobability sampling

## Abstract

Published surveys in the United States provide much evidence that COVID-19 vaccination is influenced by disease and vaccine-related risk perceptions. However, there has been little examination of whether individual’s general beliefs about vaccines are also related to COVID-19 vaccination, especially among unvaccinated adults. This study used an August 2021 national survey of 1000 U.S. adults to examine whether general beliefs about vaccines were associated with COVID-19 vaccination status. In addition, it used multivariate analyses to assess the relative contribution of individual vaccine beliefs to current vaccine status independently of COVID-19-specific attitudes and experiences, and demographics. The findings indicated that, collectively, general vaccine beliefs mattered more than demographics, COVID-19-specific risk perceptions, confidence in government, or trust in public health agencies in COVID-19 vaccination status. Overall, the findings affirm the importance of vaccine education and communication efforts that help people understand why vaccines are needed, how vaccine safety is established and monitored, and how vaccines provide protection from infectious diseases. To achieve success among vaccine-hesitant individuals, communication strategies should target vaccine beliefs that most influence vaccination outcomes.

## 1. Introduction

Sixteen months after vaccine authorization and recommendation for COVID-19, one in four adults in the United States have yet to be fully vaccinated, and one in eight remain completely unvaccinated [1]. By the end of December 2021, more than 820,000 people in the United States had died from or with COVID-19, and nearly 54 million cases had been documented [1], with infections ranging from asymptomatic to very severe illness and death [2]. Since the development and emergency-use authorization of COVID-19 vaccines in the United States in December 2020, high vaccination coverage has become the primary tool for impeding virus transmission and reducing hospitalizations and deaths. As such, COVID-19 vaccine hesitancy and refusal remain barriers to achieving the desired higher levels of vaccination [3] and are likely to be challenges as additional doses or updated vaccines are recommended in response to SARS-CoV-2 variants, such as the Omicron variant that emerged in November 2021.

National surveys during the pandemic indicated that many reasons for COVID-19 vaccination hesitancy and refusal mirror those repeatedly documented for adult influenza vaccination [4,5]. Objections to COVID-19 vaccination by U.S. adults, for instance, include not perceiving COVID-19 as a serious or significant health threat, skepticism regarding COVID-19 vaccine effectiveness, a high level of concern regarding vaccine safety or potential adverse reactions [6,7,8,9,10], and not perceiving COVID-19 vaccination benefits to outweigh known or perceived vaccination risks [11,12,13,14]. These objections have been found to vary by demographics (e.g., less prevalent among people 65 years and older and those with more years of formal education) and race/ethnicity (e.g., greater among those living in rural areas, African Americans, and those who identify as Republicans or conservative) [11,12,13,14]. Distrust in federal government agencies, public health officials, and medical authorities, along with inconsistent and changing federal, state, and local public health recommendations and messages, have likely further reduced trust in COVID-19 vaccination information and recommendations [15,16,17,18].

Overall, the published surveys related to U.S. adult COVID-19 vaccine hesitancy provide much evidence that vaccination is influenced by risk perceptions related to COVID-19 disease and vaccine, trust in government health and science agencies, and one’s demographic characteristics, including political affiliation or ideology. In general, U.S. adults who received the recommended COVID-19 vaccines perceive (and generally have) greater risk for severe COVID-19 illness, higher trust in government agencies and public health officials and information, and more confidence in the vaccines’ safety [13,14,19]. However, there has been little examination of whether one’s general beliefs about vaccines are also related to COVID-19 vaccination status, especially among unvaccinated adults.

This study provides new and important insights into the drivers of COVID-19 vaccine hesitancy and refusals by examining for the first time whether individuals’ general beliefs about vaccines are associated with, and predictive of, self-reported COVID-19 vaccination status. Moreover, this study uniquely assessed the contribution of general vaccine beliefs to COVID-19 vaccination status independently of demographics, and COVID-19-specific factors including risk of COVID-19, confidence in government agencies and experts, and trust in institutions’ information about the virus. Kessels et al. (2021) found evidence in a survey with Belgian respondents that beliefs toward vaccination in general could be associated with willingness to seek vaccination against COVID-19, but their single item measure only focused on the perceived usefulness of vaccinations to protect against infectious diseases [20].

This study involved a more extensive assessment of the influence of general vaccine beliefs, including their influence relative to demographics (including political identification), COVID-19 risk perceptions, direct experience with COVID-19 infection, and trust and confidence in government agencies and information related to COVID-19 [21]. General vaccine beliefs were assessed using ten vaccine-belief items which prove useful for understanding parents’ childhood vaccination hesitancy [22]. A query of items in the Societal Experts Action Network (SEAN) COVID-19 Survey Archive indicated virtually all measures entered to date from COVID-related surveys which include the words “vaccine” or “vaccines” focused on beliefs or behaviors specific to COVID-19 or influenza and did not involve the assessment of beliefs toward vaccines in general [23]. Little attention has been paid to whether individuals’ core beliefs toward vaccines and immunizations are associated with non-compliance with COVID-19 vaccination recommendations in the U.S. This study was thus designed to (1) identify respondents’ general beliefs towards vaccines; (2) assess whether these beliefs were associated with and predictive of their COVID-19 vaccination status; and (3) examine the relative influence of their general vaccine beliefs compared to demographics, COVID-19 risk perceptions, direct experience with COVID-19 infection, and trust and confidence in government agencies and information related to COVID-19.

## 2. Research Method

### 2.1. Participants

The survey utilized here was part of a larger research effort that involved frequent assessments of the U.S. public’s beliefs and experiences during the COVID-19 pandemic. Eleven monthly surveys were conducted during 2020 and 2021. To conduct these national surveys on such a frequent basis during the pandemic, a national nonprobability panel was used to obtain completed surveys in a rapid and cost-efficient manner [24]. Respondents for all of these surveys were drawn from the MFour mobile panel, a U.S. nonprobability market research smartphone panel, which is comprised of approximately two million persons who live in the United States.

The focus of this paper is on the last survey in this series (August 2021), which similarly to the others, was drawn as a Census-balanced national replicate sample from the panel. Individuals in the full panel own a smartphone with Android or iOS and have registered to receive and respond to survey opportunities using MFour’s Surveys On The Go^®^ app. Individuals are deemed qualified for panel inclusion following a series of profiling questions and fraud-detection measures. The panel does not provide a comprehensive population frame, or support probability samples for the general population, due to the nature of panel enrollment. However, the overall panel is designed to provide national nonprobability samples of adults that are geographically and demographically reflective of the U.S. population. The overall panel database includes the zip code, age, gender, race/ethnicity, and education of panel members so that samples can be geographically and demographically balanced to match Census estimates.

The initial survey invitation was sent by app notification via cell phone to a Census-balanced (age, gender, and race) and geographically representative national sample of approximately 4500 U.S. adult panel members. Three reminders via app push notifications were sent to non-respondents over a period of a few days. Respondents received up to a $4 incentive for participation. The self-administered web-based surveys averaged 24:17 min in length. A total of 1000 self-administered surveys were completed in the August survey, including respondents from 49 states and the District of Columbia. The survey response rate (AAPOR RR 1) was 22%, which is comparable to most other non-federal surveys of similar length [25,26] (High response rates can reduce the risk of bias in survey estimates, but research suggests that non-response bias is not necessarily associated with response rate [27]). This study was reviewed and received Institutional Review Board approval for the protection of human subjects.

### 2.2. Survey Measures

The vaccine and vaccination measures analyzed here were part of a broader survey instrument that included items related to physical and mental health, knowledge of COVID-19 symptoms, treatment-seeking for COVID-19, impact of the pandemic on employment and income, and demographics. The broader survey instrument was a standardized questionnaire with more than 100 questions included in the August version. Items were selected from the research literature or contemporary surveys where appropriate, and standard demographic questions were implemented (Appendix A). Given the broader focus, the survey invitation and introduction did not mention COVID-19 or the pandemic.

This paper uses data from the August 2021 survey, which included the following key vaccination status measures: “Have you received a COVID-19 vaccine?” and for those who responded “yes”, a follow-up question that asked how many doses they had received. COVID-19 vaccination status (any vaccination) was calculated as a “yes” response to the first question and a reply of at least one dose to the second question. (Those responding with “prefer not to answer” (29 cases) were treated as unvaccinated for the multivariate analyses).

The general vaccine or immunization-related belief measures were assessed using a scale “strongly disagree”, “somewhat disagree”, “somewhat agree” and “strongly agree” (See Table 1 for the vaccine belief items). The ten items were adapted from eleven belief items used in a national assessment of parent beliefs, intentions, and behaviors related to childhood immunization [22]. These items were introduced in the survey after the general health questions and before any items related to COVID-19 to avoid confusion with beliefs specific to COVID-19 vaccines. Later in the survey, items were introduced concerning COVID-19, including whether the respondent had ever been diagnosed with COVID-19, ever thought that they might have contracted it, were concerned about spread of the virus in the community, were concerned about new variants, whether COVID-19 was perceived as a real threat or blown out of proportion, and whether the worst was behind us. Confidence in the federal government and the U.S. Centers for Disease Control and Prevention (CDC), as well as trust in information from the federal government, health authorities, and news media (also measured using Likert scales) were also assessed. Items and the wording for these items were drawn from or adapted from existing surveys, including the Behavioral Risk Factor Surveillance Survey (BRFSS) and published polls [28]. Most of the independent variables in the risk and trust domains were measured using four-point Likert scales. Finally, demographic information was obtained, including gender, race, residence and political identification (treated as nominal variables) as well as age, education, and household income (ordinal variables). The COVID-19-specific risk perceptions, institutional confidence and trust, and demographic items used as independent variables in the regressions are included in Appendix A.

### 2.3. Statistical Analyses

A statistical analysis was conducted using the IBM SPSS Statistics 22 software. Univariate and bivariate analyses for the key dependent and independent variables described above were initially undertaken. Correlations between COVID-19 immunization and the ten belief questions were then examined. There was a relatively high rate of “not sure” responses for some of the vaccine belief items. Consequently, we conducted separate correlations by both excluding the “not sure” responses as missing values for the general vaccine belief items as well as recoding the “not sure” responses as a middle value between the agree and disagree values. Since we found little difference in correlations between the two approaches, but a substantial loss in the number of cases if “not sure” was treated as a missing value, we conducted subsequent analyses using the vaccine items with “not sure” recoded to a middle point on the agreement scale.

We conducted linear regression analyses with vaccine status as the dependent variable and the individual vaccine beliefs as independent predictors. We chose linear regression rather than logistic regression for this paper because the R square value as a measure of percentage of variance explained by the model is a helpful tool for many readers in evaluating the models. Similarly, the standardized beta provides an easy interpretation of the rank order of influence for the independent variables in the model. Finally, since the key independent variables and general vaccine beliefs are ordinal measures, we did not have to reduce the potential explanatory power of these variables by converting them to dichotomous measures in a logistic regression. The standardized beta weight indicates the amount of change in one standard deviation in the dependent variable associated with this amount of change in the standard deviation of the independent variable.

In the first model, we introduced only the vaccine belief items as independent variables to predict COVID-19 vaccination status. Then, in a second model, we included independent variables from the demographic, COVID-19 specific risk measures and attitudes toward institutions, as well as general vaccine beliefs domains in the linear regression on COVID-19 vaccination status. The incremental, stepwise introduction of general immunization beliefs along with potential predictors of vaccination status from the other domains allowed us to conduct an assessment of the magnitude of each domain’s contribution to the variance explained in the second model. Since the dependent variable of COVID-19 vaccination status was coded as 0 (no) and 1 (yes) and the independent vaccine belief variables were coded from 1 (strongly disagree) to 5 (strongly agree), a positive correlation or standardized beta would indicate that agreement was positively related to vaccination.

## 3. Results

A total of 1000 people participated in the August 2021 national survey. Post-stratification weights were calculated and applied to the achieved sample by raking Census estimates of age, gender, and race/ethnicity based on 2015–2019 ACS data. Raking is an iterative form of post-stratification that allows for adjustments to additional variables and categories, and is especially effective for non-probability samples as described, for example, in Iachan et al. (2019) [29]. The general characteristics of both the weighted and unweighted sample are presented in Appendix B.

Weighted estimates are provided in the vaccine beliefs presentation (Table 1), but the correlation and regression data are unweighted. The survey variables used in the survey for the vaccine belief questions are included in Table 1 alongside the wording of questions (VacA to VacK). Although it was not the purpose of this research to estimate vaccination rates, the findings are consistent with those from other published polls during this period [15,16,17] and CDC’s COVID-19 Tracker estimates (The proportion who reported that they had received at least one dose of COVID-19 vaccine was 68% of the answering respondents and 66% of all respondents, compared to CDC’s estimate of 71% of all US adults on 9 August 2021, while the proportion who reported receiving both doses of a two-dose vaccine or a single dose of a one-dose vaccine and thus were fully vaccinated was 63% in the survey, the same as the CDC estimate on the first day of the field period.).

### 3.1. General Vaccine-Related Beliefs

The majority of respondents in the survey held positive general beliefs about vaccines. Nearly three quarters or more agreed that vaccines overall were very effective (78%), that vaccines were important to their health (77%), being vaccinated was important to the health of the others in the community (75%) and that overall, vaccines are very safe (73%). However, a substantial proportion of respondents held beliefs that may generate reluctance or unwillingness to receive vaccines, particularly newly recommended vaccines. While a majority agreed that there was little risk in getting the disease from a vaccine (57%) and that information about vaccines from government health agencies was reliable and trustworthy (56%), four of ten respondents did not agree with these statements (Table 1). In addition, a majority (53%) believed that some vaccines have ingredients that may be harmful, 40% believed some vaccines were linked to long-term health problems, 31% perceived some risk of getting the disease from the vaccine, and 26% agreed that some vaccines may cause learning disabilities, such as autism. Further, 32% agreed that natural infection was safer than vaccines for providing immunity.

A VacF question was not included in the survey.

### 3.2. Correlation of Immunization Beliefs and COVID-19 Vaccination Status

Bivariate correlations were used as an initial step in examining relationships between general vaccine beliefs and COVID-19 vaccination status. When “not sure” responses were excluded, all ten general beliefs about vaccines were moderately to strongly correlated with COVID-19 vaccination status (p’s < 0.001) (Table 2). Correlations ranged from 0.389 for the belief that there was little risk of getting the disease from a vaccine to 0.588 for the belief that being vaccinated was important for the health of others in the community.

Although some of the general vaccine belief items produced only a modest amount of “not sure” responses (i.e., 6–8%), six produced a proportion of between 10% and 27% “not sure” responses. Although “not sure” may be a meaningful response category for some analyses as well as an accurate reflection of respondents’ understanding, it creates problems for the use of ordinal variables in correlation and regression analyses. Consequently, they are frequently treated as missing values in these types of analyses. Unfortunately, treating this high proportion of “not sure” responses in six vaccine belief measures as missing values would require a substantial loss in the number of otherwise valid cases in subsequently analyses, or a loss of important vaccine belief items. One frequently used alternative is to recode “missing values” to a mean or midpoint on the scale [30]. In the analyses that included these responses, the “not sure” responses were recoded to the middle of the scale (3) between disagree (1, 2) and agree (4, 5) in a second set of vaccine belief analyses. This approach resulted in very little difference in most correlations, ranging from −0.352 for beliefs that some vaccines may cause learning disabilities to 0.588 for the belief that being vaccinated is important for the health of others, and no difference in statistical significance (Table 2). Consequently, the subsequent correlation and multivariate analyses used general vaccine belief measures with “not sure” recoded to a middle position on the agreement scale.

Before conducting regression analyses to determine the significant predictors of COVID-19 vaccination status, we conducted bivariate correlations among the ten general vaccine measures (with not sure included but recoded to the midpoint). The positive vaccine beliefs were positively correlated with each other, negatively correlated with negative beliefs, and vice versa. The size of the correlations ranges from 0.290 to 690. Thirty-two out of the forty-five bivariate correlations were less than 0.50 (Table 3). This moderate level of correlation encouraged us to enter each of the individual beliefs as potential predictors of COVID-19 vaccination status in subsequent regressions. A factor analysis (not shown in the tables) was also performed to ensure that there were no underlying dimensions of general vaccine beliefs that would act as more informative predictors of vaccination status than the individual items, but the only components that emerged were a positive belief one and a negative belief one.

### 3.3. Predicting COVID-19 Vaccination Status with General Beliefs about Vaccines

As noted, vaccination status was based on two responses—the respondent indicating receipt of a COVID-19 vaccine and reporting at least one dose received. In a linear multiple regression analysis, the ten general beliefs about vaccines were regressed on COVID-19 vaccination status with “not sure” responses for vaccine beliefs recoded to a middle position on the agreement scale. Six of the ten general vaccine beliefs had a significant relationship with vaccination status. Three had a significant positive relationship: there is little risk of getting the disease from a vaccine (0.071), vaccines are very effective (0.097), and my being vaccinated is important for the health of others in the community (0.378). Conversely, the beliefs that natural infection is safer than vaccines (−0.106), some vaccines are linked to long term health problems (−0.082), and some vaccines have ingredients that could be harmful (−0.083) were significant negative predictors of COVID-19 vaccination status. The R square for the regression model was (0.403) (Table 4).

### 3.4. Combined Regression of Demographic, COVID-19 Specific and General Vaccine Beliefs

The demographic, COVID-19 specific risks, attitudes toward institutions, and general vaccine beliefs domains were combined as independent variables in a subsequent linear multiple regression model designed to predict COVID-19 vaccination status. In this model, four demographic variables were associated with vaccination status: household income (0.081), age (0.053), education (0.080), and not Black or African American race (0.051). Only one of the ten COVID-19-specific risk or trust items—concern about new variants of the virus (0.105)—was associated with vaccination status. Notably, however, four general vaccine beliefs were significant predictors of COVID-19 vaccination status in this model when entered with demographics and COVID-19 belief variables. Two general vaccine beliefs were negatively associated with COVID-19 vaccination status (natural infection is safer than vaccines for providing immunity (−0.107), and some vaccines have ingredients that could be harmful (−0.115)), while two were positively associated with COVID-19 vaccination status (vaccines are very effective (0.090), and my being vaccinated is important for the health of others in the community (0.366)). Altogether, nine of the measures provided independent contributions to the second model for COVID-19 vaccination status. The second model achieved a somewhat higher predictive value (0.428) with nearly the full sample (*N* = 942) in the model (Table 5).

## 4. Discussion

Despite the widespread availability of safe and effective COVID-19 vaccines at no cost to individuals, one in four adults in the United States were not fully vaccinated sixteen months after vaccinations had been approved and recommended. Strong vaccine hesitancy and refusal thus remain significant contributors to COVID-19 infections and illnesses in the U.S. and more importantly, hospitalizations and deaths [31,32,33,34,35]. As such, it is vital to continue to identify and assess potential factors behind adult non-compliance with COVID-19 vaccination recommendations.

This study provides new and important insights into the drivers of COVID-19 vaccine hesitancy and refusals by examining for the first time whether individuals’ general beliefs about vaccines are associated with, and predictive of, self-reported COVID-19 vaccination status. Moreover, this study uniquely assesses the contribution of general vaccine beliefs to COVID-19 vaccination status independently of demographics, and COVID-19-specific factors including risk of COVID-19, confidence in government agencies and experts, and trust in institutions’ information about the virus.

In line with much previous COVID-19 vaccination acceptance research [14,36,37], the findings here confirm that demographic characteristics, particularly age, education, household income, and not being Black, are associated with uptake and declination. More importantly, this study found that adults’ general (i.e., existing) beliefs about vaccines are correlated with, and predictive of, COVID-19 vaccination status. For at least 20 years, much research related to recommended infant and childhood immunization acceptance has focused on and found that parents’ general beliefs regarding vaccines and vaccination often matter [38,39]. In line with those findings, our study found evidence for the first time that adults’ existing beliefs regarding vaccines likely contribute to COVID-19 vaccination hesitancy and refusal among a significant portion of the adult population.

Our findings affirmed the importance of fostering and maintaining positive beliefs and understanding the value and benefits of vaccines among not just parents, but among the broader adult population. Existing vaccine beliefs matter for both those who have accepted COVID-19 vaccination and those who have not. Indeed, collectively, general or existing vaccine beliefs may matter as much or more than demographics, COVID-19-specific risk perceptions, and confidence in government and trust in public health agencies, with respect to COVID-19 vaccination status. Much effort is directed towards educating parents about the effectiveness, benefits, and safety of recommended childhood immunization, and the findings here illustrate how important positive beliefs regarding vaccines are when it comes to adult vaccination acceptance. In this study, COVID-19 vaccination status was strongly associated with believing that vaccines are effective, safe, and important for protecting both individual health and the health of others in the community. Notably, the most powerful predictor of COVID-19 vaccination is the belief that being vaccinated is important for the health of others in the community.

Conversely, COVID-19-unvaccinated individuals held beliefs that reflected skepticism and distrust of vaccines more broadly. In addition, respondents who had not yet received a COVID-19 vaccination held beliefs similar to parents who are strongly hesitant or resistant to recommended childhood vaccinations. Notably, this included believing that natural infection was safer than vaccination, some vaccine ingredients could be harmful, and vaccines could cause long-term health problems. The implications are two-fold. Firstly, it is essential that efforts to foster COVID-19 vaccination among unvaccinated adults recognize the existence and likely the deep influence of general beliefs about vaccines. This includes recognition that vaccination education efforts will likely require time, patience, and approaches that seek to build understanding beyond COVID-19 vaccines. This is not to say vaccine education outreach should not also address vaccine concerns that are specific to the COVID-19 pandemic and COVID-19 vaccines. Rather, the findings of this study suggest that individual’s general beliefs about vaccines are potentially the most powerful modifiable factors influencing COVID-19 vaccination and suggest that vaccine education efforts should be targeted to vaccine beliefs that most influence vaccination outcomes. Second, given that many of the negative beliefs about vaccines are long held, this study’s findings illustrate the need for greater and earlier vaccine education efforts. It is likely that many adults who have not yet received a COVID-19 vaccination will be difficult to reach with vaccine education or may not be willing to partake in vaccine education efforts. Efforts targeting middle and high school students as well as young adults would likely be more useful.

Additionally, the findings from the regression analyses with demographics, general vaccine beliefs and COVID-19-related experiences and perceptions are notable. Overall, these analyses suggested that many COVID-19 specific factors may have relatively little association with COVID-19 vaccination status when demographics and general vaccine beliefs are taken into account. For example, having been diagnosed with COVID-19 or having been sick and thinking you might have the virus were not significant predictors of immunization. Similarly, concerns about COVID-19 spread in one’s community or believing the worst of the pandemic was still ahead of us were not significant predictors of one’s COVID-19 vaccination status. Finally, this study did not find that confidence in the federal government or CDC, or trust in the information from health experts or the media, were significantly related to COVID-19 vaccination status. Only concerns about new variants of the COVID-19 virus were associated with COVID-19 vaccination status when demographic and general vaccine beliefs were included in the model. It should be noted, however, that some published studies, such as that of Yang et al., 2022, found that components of the Health Belief Model, which places emphasis on risk-benefit perceptions, perceived barriers, and cues to action, have found some associations with COVID-19 vaccination (e.g., perceived benefits, information sources used) [40].

Regardless of the role that these findings can play in the resolution of the COVID-19 pandemic, these results have important implications for planning for future outbreaks of communicable diseases for which vaccines are available. The findings here suggest public health strategies will need to address—and overcome—the broader vaccine-related beliefs of COVID-19 vaccination resisters. This includes recognizing that many of those yet to receive a COVID-19 vaccination have questions and concerns that encompass more than COVID-19 vaccines. These findings also suggest that the COVID-19-specific context of a highly politicized environment, polarized beliefs about the risk of the disease and trust in public health agencies, rapid vaccine development, novel vaccine models, and vaccine specific side effect concerns, had less to do with the eventual adoption of the vaccines than existing beliefs about vaccines and vaccination in general. If this is true, then public health leaders need to meaningfully invest in communications strategies that can modify or ameliorate general vaccine beliefs that present barriers to future immunization efforts.

## 5. Limitations

This study used a demographically and geographically representative non-probability sample from a large, national consumer panel to assess immunization-related beliefs and vaccine behaviors during the pandemic. A limitation of non-probability samples is that projections of estimates to the broader population within statistical limits are not possible, unlike with probability samples. However, the survey estimates from non-probability samples are not necessarily biased or unrepresentative. Moreover, the primary focus of this study was on the correlates and predictors of vaccine acceptance in the population, not point estimates of the population. Thus, while probability samples remain the gold standard for survey research, non-probability samples are increasingly accepted when factors such as a limited budget, high data collection costs, or urgency make it infeasible to use a probability sample. Indeed, the CDC has actively conducted national non-probability web surveys to measure vaccine beliefs and behaviors related to seasonal flu for a decade [41,42]. Although a non-probability sample is a limitation, we believe that this study meets the “fit-for-purpose” criteria for survey design, particularly when there are no probability samples providing equivalent data.

A second limitation of the study is that the analysis is based on the Health Belief Model which posits that health behaviors are the result of one’s perceptions of risk susceptibility, severity of disease, perceived benefits of acting, cues to action, and information sources. While this study did not find COVID-19 vaccine-related risk-benefit perceptions associated with vaccination status, those perceptions are likely to change over the course of a pandemic. A cross-sectional survey, with a probability or a non-probability sample, cannot collect information on changes over time and can only demonstrate association as opposed to causation between beliefs and behaviors.

## 6. Conclusions

This study found that general vaccine beliefs may be an overlooked influence when it comes to U.S. adults’ COVID-19 vaccination behaviors. Indeed, this study suggests that general vaccine beliefs contribute more to COVID-19 vaccination status than demographic characteristics, COVID-19 specific risk concerns, and attitudes toward government and public health institutions. Second, this study provided important insights into COVID-19 vaccination hesitancy and refusal, including those of the general vaccine beliefs held by COVID-19 non-vaccinators at present which will be very difficult to overcome. These are likely long-held beliefs based on limited actual knowledge or understandings of how vaccines work or why they are recommended. In addition, frameworks such as the Health Belief Model, while useful for calling attention to the influence of disease susceptibility and severity perceptions on behavior, offer little in terms of how to influence people who strongly do not perceive a meaningful health threat. Finally, the importance of general vaccination beliefs in predicting COVID-19 vaccination status, compared to other COVID-19-specific attitudes and experiences, suggests that these findings will be relevant to public health efforts when managing vaccination hesitancy and refusal in future epidemics.

## Figures and Tables

**Table 1:** General beliefs about vaccines: August 2021 (weighted).

Vac. Please Indicate How Much You Would Agree or Disagree with the Following Statements about Vaccines, in General.							
	*N*	Strongly Disagree	Somewhat Disagree	Somewhat Agree	Strongly Agree	Not Sure	NA
**VacA.** Some vaccines are linked to long term health problems.	1000	18.8%	17.0%	28.1%	12.1%	23.9	0.1
**VacB.** Natural infection is safer than vaccines for providing immunity.	1000	28.3%	21.3%	20.5%	11.2%	18.5%	0.2
**VacC.** There is little risk of getting the disease from the vaccine.	1000	12.2%	18.9%	26.4%	30.4%	12.1%	-
**VacD.** Some vaccines may cause learning disabilities, such as autism.	1000	31.2%	15.6%	16.7%	9.3%	26.9%	0.3
**VacE.** Some vaccines have ingredients that could be harmful.	1000	13.8%	13.7%	36.4%	16.4%	19.3%	0.3
**VacG.** Overall, vaccines are very safe.	1000	7.0%	11.2%	36.3%	36.7%	8.5%	0.3
**VacH.** Overall, vaccines are very effective.	1000	4.8%	9.6%	36.3%	42.1%	7.0%	0.2
**VacI.** Vaccines are important for my health.	1000	6.6%	10.3%	33.6%	43.5%	5.8%	0.2
**VacJ.** My being vaccinated is important for the health of others in my community.	1000	8.4%	10.3%	26.2%	48.4%	6.3%	0.5
**VacK.** The information I receive about vaccines from government health agencies is reliable and trustworthy	1000	15.8%	18.5%	33.6%	22.1%	9.6%	0.4

There was not a VacF question included in the survey.

**Table 2:** Beliefs about vaccines and Received COVID-19 Vaccine.

VAC1a. Have You Received a COVID-19 Vaccine? VAC1b. Have You Received the First Dose? VACCINE = VAC1a EQ Yes and VAC1b NE No Excludes Prefer Not to Answer						
BELIEFS Vac. Please indicate How Much You Would Agree or Disagree with the Following Statements about Vaccines, in General.	VACCINE (Attitudes Exclude Not Sure)			VACCINE (Attitudes Recode Not Sure to Middle of the Scale)		
	*N*	R	Sign.	*N*	R	Sign.
**VacA.** Some vaccines are linked to long term health problems.	762	−0.455	0.001	999	−0.412	0.001
**VacB.** Natural infection is safer than vaccines for providing immunity.	812	−0.449	0.001	998	−0.411	0.001
**VacC.** There is little risk of getting the disease from the vaccine.	870	0.389	0.001	1000	0.362	0.001
**VacD.** Some vaccines may cause learning disabilities, such as autism.	720	−0.422	0.001	997	−0.352	0.001
**VacE.** Some vaccines have ingredients that could be harmful.	798	−0.416	0.001	996	−0.380	0.001
**VacG.** Overall, vaccines are very safe.	908	0.495	0.001	997	0.482	0.001
**VacH.** Overall, vaccines are very effective.	928	0.484	0.001	998	0.473	0.001
**VacI.** Vaccines are important for my health.	941	0.478	0.001	998	0.478	0.001
**VacJ.** My being vaccinated is important for the health of others in my community.	931	0.588	0.001	995	0.588	0.001
**VacK.** The information I receive about vaccines from government health agencies is reliable and trustworthy	896	0.464	0.001	997	0.451	0.001

**Table 3:** Correlations among General Beliefs about Vaccines (UNWEIGHTED).

Not Sure Responses Recoded to Midpoint in Scale (Prefer Not to Answer Excluded)										
	VACA	VACB	VACC	VACD	VACE	VACG	VACH	VACI	VACJ	VACK
	(999)	(998)	(1000)	(997)	(996)	(997)	(998)	(998)	(995)	(997)
VACA	1.00									
VACB	0.473	1.00								
VACC	−0.326	−0.290	1.00							
VACD	0.628	0.471	−0.328	1.00						
VACE	0.606	0.408	−0.304	0.513	1.00					
VACG	−0.437	−0.414	0.455	−0.448	−0.410	1.00				
VACH	−0.421	−0.395	0.459	−0.421	−0.373	0.690	1.00			
VACI	−0.411	−0.412	0.399	−0.413	−0.341	0.649	0.687	1.00		
VACJ	−0.443	−0.458	0.438	−0.411	−0.383	0.635	0.619	0.683	1.00	
VACK	−0.475	−0.381	0.428	−0.418	−0.436	0.600	0.553	0.596	0.604	1.00

All correlations are significant at 0.01 level (2 tailed).

**Table 4:** Predicting COVID-19 Vaccines Received by General Beliefs toward vaccines (unweighted).

INDEPENDENT VARIABLES Vac. Please Indicate How Much You Would Agree or Disagree with the Following Statements about Vaccines, in General.	Received COVID-19 VACCINE, Missing Values on Vaccine Attitudes Recoded to Middle		
	*N* = 979	R^2^ = 0.403	Standardized Beta (Only if Significant at 0.05)
**VacA.** Some vaccines are linked to long term health problems			−0.082
**VacB.** Natural infection is safer than vaccines for providing immunity			−0.106
**VacC.** There is little risk of getting the disease from the vaccine			0.071
**VacD.** Some vaccines may cause learning disabilities like autism			
**VacE.** Some vaccines have ingredients that could be harmful			−0.083
**VacG.** Overall vaccines are very safe			
**VacH.** Overall vaccines are very effective			0.097
**VacI.** Vaccines are important for my health			
**VacJ.** My being vaccinated is important for the health of others in my community			0.378
**VacK.** The information I receive about vaccines from government health agencies is reliable and trustworthy			

**Table 5:** Predicting COVID-19 Vaccine Received by Demographics, COVID-19 Specific Factors, and General Vaccine Beliefs.

	Received COVID-19 VACCINE, Missing Values on Vaccine Attitudes Recode to Middle		
	*N* = 942	R^2^ = 0.428	Standardized Beta (Only if Significant at 0.05)
**INDEPENDENT VARIABLES**			
Household Income			0.081
Gender			
Age			0.053
Hispanic			
Not White			
Not Black			0.051
Education			0.080
Democrat			
Republican			
Suburbs			
Rural			
Real threat			
Concerned about new variants			0.105
Thought I had virus			
Diagnosed with COVID-19			
Concerned about community spread			
Worst is behind us			
Important to wear face mask			
Confidence in federal government			
Confidence in CDC			
Trust information from health experts			
Trust info from news media			
**VacA.** Some vaccine are linked to long term health problems			
**VacB.** Natural infection is safer than vaccines for providing immunity			−0.107
**VacC.** There is little risk of getting the disease from the vaccine			
**VacD.** Some vaccines may cause learning disabilities like autism			
**VacE.** Some vaccines have ingredients that could be harmful			−0.115
**VacG.** Overall vaccines are very safe			
**VacH.** Overall vaccines are very effective			0.090
**VacI.** Vaccines are important for my health			
**VacJ.** My being vaccinated is important for the health of others in my community			0.366
**VacK.** The information I receive about vaccines from government health agencies is reliable and trustworthy			

## Data Availability

Not applicable.

## Appendix A. Survey Variables Used in Regression Models

		**Codes**	**Not Selected**
**Gender**: What is your gender?			
	Male	1	
	Female	2	
**Age**: What is your age?			
	18–24	1	
	25–34	2	
	35–49	3	
	50–64	4	
	65+	5	
**Education:** What is the highest grade or year of school you completed?			
	Less than High School Degree	1–3	
	High School Graduate/GED	4	
	Some College	5	
	College Graduate	6	
**Household Income:** From panel profile			
	Less than $25,000	1	
	$25,000 to $34,999	2	
	$35,000 to $49,999	3	
	$50,000 to $74,999	4	
	$75,000 to $99,999	5	
	$100,000 or more	6	
**Hispanic:** Are you Hispanic, Latino or Spanish origin?			
	Yes	1	
	No	2	
**Race:** Which one or more of the following would you say is your race?			
	White	1	2
	Black or African/American	1	2
	American Indian or Alaskan Native	1	2
	Asian	1	2
	Pacific Islander	1	2
	Other	1	2
	Refused	1	2
**Political Affiliation**: Generally speaking do you think of yourself as a …			
	Democrat	1	0
	Republican	1	0
	Independent	1	0
	Something else	1	0
Do you live in			
	City	1	0
	Suburb	1	0
	Rural area	1	0
**Real threat**: Do you think the coronavirus (COVID-19) is a real threat or blown out of proportion?			
	Real threat	1	0
How concerned are you about **new variants** of COVID-19, like the delta variant?			
	Extremely, moderately, slightly, not at all	1,2,3,4	
Since the beginning of the coronavirus (COVID-19) crisis in late January 2020, have you had a period of 3 days or longer when you were sick and **thought you might have the coronavirus** (COVID-19)?			
	Yes	1	
	No	2	
**Diagnosed**: Have any of the following household members been diagnosed as having coronavirus (COVID-19)?			
	Yes, I have/Yes, both myself and at least one other person in the my household	1	
	Yes someone else in my household/No one in household	0	
Are you very concerned, concerned, or not very concerned about the **spread of coronavirus** (COVID-19) in your community?			
	Very concerned, concerned, not very concerned	1,2,3	
How important do you think it is for people like you to do the following in order to stop the spread of the coronavirus (COVID-19)? **Wearing a face mask**			
	Very, Somewhat, Not too, it should not be done	4,3,2,1	
How much confidence do you have in the following organizations to deal with the outbreak of the coronavirus (COVID-19)? **Federal government** **Centers for Disease Control (CDC)**			
	Great deal, fair amount, not very much, no confidence at all	4,3,2,1	
Do you trust the information you hear about the coronavirus (COPVID-19) from the following organizations? **Public health experts** **New Media**			
	Great deal, fair amount, not very much, no confidence at all	4,3,2,1	
**Worst is yet to come**: Which of the following best describes your feelings about the coronavirus (COVID-19) in the United States?			
	The worst is yet to come	1	
	The worst is behind us, the coronavirus is not likely to be that major of a problem	0	

## Appendix B. Demographic Characteristics of the Sample

		**Unweighted**	**Weighted**
**Gender**			
*N* = 995	Male	40.1%	48.6%
	Female	59.9%	51.4%
**Age**			
*N* = 1000	18–24	9.0%	12.2%
	25–34	17.1%	17.9%
	35–49	27.7%	25.6%
	50–64	30.4%	24.5%
	65+	15.8%	19.7%
**Education**			
*N* = 995	Less than High School Degree	9.0%	5.5%
	High School Graduate/GED	23.9%	23.8%
	Some College	35.4%	35.8%
	College Graduate	35.1%	34.9%
**Household Income**			
*N* = 1000	Less than $25,000	24.6%	25.2%
	$25,000 to $34,999	13.4%	12.9%
	$35,000 to $49,999	16.4%	16.6%
	$50,000 to $74,999	17.9%	18.0%
	$75,000 to $99,999	12.1%	11.9%
	$100,000 or more	15.6%	15.5%
**Hispanic, Latino/a or Spanish origin**			
*N* = 1000	Yes	18.5%	15.9%
	No	81.5%	84.1%
**Race**			
*N* = 1000	White	77.5%	74.6%
	Black or African/American	10.4%	13.5%
	American Indian or Alaskan Native	3.3%	3.4%
	Asian	3.9%	4.3%
	Pacific Islander	0.8%	0.8%
	Other	6.6%	6.0%
	Refused	0.8%	0.6%
**Census Division**			
*N* = 1000	New England	3.6%	3.2%
	Middle Atlantic	15.3%	14.0%
	East North Central	12.9%	14.1%
	West North Central	6.7%	6.8%
	South Atlantic	19.8%	20.0%
	East South Central	6.3%	6.2%
	West South Central	12.3%	11.9%
	Mountain	8.2%	8.3%
	Pacific	14.9%	15.4%

## References

[1] Coronavirus in the U.S.: Latest Map and Case Count. The New York Times.

[2] CDC (2021). Interim Clinical Guidance for Management of Patients with Confirmed Coronavirus Disease (COVID-19). https://www.cdc.gov/coronavirus/2019-ncov/hcp/clinical-guidance-management-patients.html.

[3] Fisk R.J. (2021). Barriers to vaccination for coronavirus disease 2019 (COVID-19) control: Experience from the United States. Glob. Health J..

[4] Schmid P., Rauber D., Betsch C., Lidolt G., Denker M.-L. (2017). Barriers of Influenza Vaccination Intention and Behavior–A Systematic Review of Influenza Vaccine Hesitancy 2005–2016. PLoS ONE.

[5] Quinn S.C., Jamison A.M., An J., Hancock G.R., Freimuth V.S. (2019). Measuring vaccine hesitancy, confidence, trust and flu vaccine uptake: Results of a national survey of White and African American adults. Vaccine.

[6] Pomara C., Sessa F., Ciaccio M., Dieli F., Esposito M., Giammanco G.M., Garozzo S.F., Giarratano A., Prati D., Rappa F. (2021). COVID-19 Vaccine and Death: Causality Algorithm According to the WHO Eligibility Diagnosis. Diagnostics.

[7] Pomara C., Sessa F., Ciaccio M., Dieli F., Esposito M., Garozzo S.F., Giarratano A., Prati D., Rappa F., Salerno M. (2021). Post-mortem findings in vaccine-induced thrombotic thombocytopenia. Haematologica.

[8] Sessa F., Salerno M., Esposito M., Di Nunno N., Zamboni P., Pomara C. (2021). Autopsy Findings and Causality Relationship between Death and COVID-19 Vaccination: A Systematic Review. J. Clin. Med..

[9] Mungmunpuntipamtip R., Wiwanitkit V. (2021). Deaths associated with newly launched SARS-CoV-2 vaccination. Leg. Med. (Tokyo).

[10] Pomara C., Salerno M., Esposito M., Sessa F., Certo F., Tripodo C., Rappa F., Barbagallo G.M. (2022). Histological and immunohistochemical findings in a fatal case of thrombotic thrombocytopenia after Chadox1 nCov-19 vaccination. Pathol.-Res. Pract..

[11] Hopkins J.S. (2020). Ahead of COVID-19 Vaccine, Half of Americans Indicate Reluctance, WSJ/NBC Poll Finds. Wall Street Journal (Eastern Ed.). https://www.wsj.com/articles/ahead-of-covid-19-vaccine-half-of-americans-indicate-reluctance-wsj-nbc-poll-finds-11602734460.

[12] Reiter P.L., Pennell M.L., Katz M.L. (2020). Acceptability of a COVID-19 vaccine among adults in the United States: How many people would get vaccinated?. Vaccine.

[13] Beleche T., Ruhter J., Kolbe A., Marcus J., Bush L., Sommers B. (2021). COVID-19 Vaccine Hesitancy: Demographic Factors, Geographic Patterns, And Changes Over Time.

[14] Daly M., Jones A., Robinson E. (2021). Public trust and willingness to vaccinate against COVID-19 in the U.S. from October 14, 2020, to March 29, 2021. JAMA.

[15] Boyon N. (2020). Three in Four Adults Globally Say They Would Get a Vaccine for COVID-19. https://www.ipsos.com/en-us/news-polls/WEF-covid-vaccine-global.

[16] O’Keefe S.M. (2020). One in Three Americans Would Not Get COVID-19 Vaccine. https://news.gallup.com/poll/317018/one-three-americans-not-covid-vaccine.aspx.%20Accessed%2012%20September%202020.

[17] Thigpen C.L., Funk C. (2020). Most Americans Expect a COVID-19 Vaccine within a Year; 72% Say They Would Get Vaccinated. https://www.pewresearch.org/fact-tank/2020/05/21/most-americans-expect-a-covid-19-vaccine-within-a-year-72-say-they-would-get-vaccinated/.

[18] Nadeem R. (2021). Growing Share of Americans Say They Plan to Get a COVID-19 Vaccine—Or Already Have. https://www.pewresearch.org/science/2021/03/05/growing-share-of-americans-say-they-plan-to-get-a-covid-19-vaccine-or-already-have/.

[19] Kaiser Family Foundation (2021). Does the Public Want to Get A COVID-19 Vaccine? When?: An Ongoing Research Project Tracking the Public’s Attitudes and Experiences with COVID-19 Vaccinations. https://www.kff.org/coronavirus-covid-19/dashboard/kff-covid-19-vaccine-monitor-dashboard/..

[20] Kessels R., Luyten J., Tubeuf S. (2021). Willingness to get vaccinated against COVID-19 and attitudes toward vaccination in general. Vaccine.

[21] Steel Fisher G.K., Blendon R.J., Caporello H. (2021). An Uncertain Public—Encouraging Acceptance of COVID-19 Vaccines. N. Engl. J. Med..

[22] Boyle J., Berman L., Nowak G.J., Iachan R., Middleton D., Deng Y. (2020). An assessment of parents’ childhood immunization beliefs, intentions, and behaviors using a smartphone panel. Vaccine.

[23] Aizpurua E. (2020). Interview the Expert: The Societal Experts Action Network (SEAN) COVID-19 Survey Archive, with Gary Langer. Surv. Pract..

[24] Baker R., Brick J.M., Bates N.A., Battaglia M., Couper M.P., Dever J.A., Gile K.J., Tourangeau R. (2013). Summary Report of the AAPOR Task Force on Non-probability Sampling. J. Surv. Stat. Methodol..

[25] California Health Interview Survey (2020). CHIS 2019 Methodology Series: Report 4-Response Rates.

[26] (2019). Survey Response Rates: Rapid Literature Review. https://militaryfamilies.psu.edu/wp-content/uploads/2019/12/Survey_Response_Rates.pdf.

[27] Centers for Disease Control and Prevention (2021). CDC-BRFSS-Questionnaires. https://www.cdc.gov/brfss/questionnaires/index.htm..

[28] Groves R., Peytcheva E. (2008). The Impact of Nonresponse Rates on Nonresponse Bias: A Meta-Analysis. Public Opin. Q..

[29] Iachan R., Berman L., Kyle T.M., Martin K.J., Deng Y., Moyse D.N., Middleton D., Atienza A. (2019). Weighting Non-probability and Probability Sample Surveys in Describing Cancer Catchment Areas. Cancer Epidemiol. Prev. Biomark..

[30] Raaijmakers Q.A.W. (1999). Effectiveness of Different Missing Data Treatments in Surveys with Likert-Type Data: Introducing the Relative Mean Substitution Approach. Educ. Psychol. Meas..

[31] Rittle C. (2022). COVID-19 Vaccine Hesitancy and How to Address It. Workplace Health Saf..

[32] Puri N., Coomes E.A., Haghbayan H., Gunaratne K. (2020). Social media and vaccine hesitancy: New updates for the era of COVID-19 and globalized infectious diseases. Hum. Vaccines Immunother..

[33] Sallam M. (2021). COVID-19 Vaccine Hesitancy Worldwide: A Concise Systematic Review of Vaccine Acceptance Rates. Vaccines.

[34] Dror A.A., Eisenbach N., Taiber S., Morozov N.G., Mizrachi M., Zigron A., Srouji S., Sela E. (2020). Vaccine hesitancy: The next challenge in the fight against COVID-19. Eur. J. Epidemiol..

[35] Biswas N., Mustapha T., Khubchandani J., Price J.H. (2021). The Nature and Extent of COVID-19 Vaccination Hesitancy in Healthcare Workers. J. Community Health.

[36] Hill P.L., Burrow A.L., Strecher V.J. (2021). Sense of purpose in life predicts greater willingness for COVID-19 vaccination. Soc. Sci. Med..

[37] Kelly B.J., Southwell B.G., McCormack L.A., Bann C.M., MacDonald P.D., Frasier A.M., Bevc C.A., Brewer N.T., Squiers L.B. (2021). Predictors of willingness to get a COVID-19 vaccine in the U.S. BMC Infect. Dis..

[38] Gellin B.G., Maibach E.W., Marcuse E.K. (2000). Do Parents Understand Immunizations? A National Telephone Survey. Pediatrics.

[39] Diaz Crescitelli M.E., Ghirotto L., Sisson H., Sarli L., Artioli G., Bassi M.C., Appicciutoli G., Hayter M. (2020). A meta-synthesis study of the key elements involved in childhood vaccine hesitancy. Public Health.

[40] Yang X., Wei L., Liu Z. (2022). Promoting COVID-19 vaccination: Does information acquisition from divergent sources make a difference?. Int. J. Environ. Res. Public Health.

[41] CDC (2011). Influenza vaccination among pregnant women—United States, 2010–2011 influenza season. MMWR.

[42] CDC (2012). Influenza vaccination coverage among health-care personnel—The 2011–12 season, United States. MMWR.

